# Acute traumatic abdominal wall hernia—value of the physical examination: case report

**DOI:** 10.1093/jscr/rjab314

**Published:** 2021-07-23

**Authors:** Barbara Yordanis Hernandez Cervantes, Duniesky Martínez Lopez, Radisnay Guzman Lambert, Mariuska Rodríguez Gonzalez, Mary Meah

**Affiliations:** Department of Surgery, School of Medicine, University of Health and Allied Sciences, Ho, Volta Region, Ghana; Department of Internal Medicine, School of Medicine, University of Health and Allied Sciences, Ho, Volta Region, Ghana; Department of Surgery, School of Medicine, University of Health and Allied Sciences, Ho, Volta Region, Ghana; Department of Internal Medicine, School of Medicine, University of Health and Allied Sciences, Ho, Volta Region, Ghana; Department of Surgery, School of Medicine, University of Health and Allied Sciences, Ho, Volta Region, Ghana

## Abstract

Acute traumatic abdominal wall hernia (TAWH) is a rare type of hernia that occurs after a low or high velocity impact of the abdominal wall against a blunt object with few cases reported. Perforations of the hollow viscera commonly follow abdominal trauma and likely require surgery for hemorrhage and sepsis source control. We report a case where a high velocity impact of the abdominal wall against the stump of a felled tree caused a TAWH with concomitant gastric perforation in a 20-year-old male patient who required exploratory laparotomy with primary repair of the stomach and fascia. The physical examination findings without previous history of abdominal hernia and pneumoperitoneum in the chest X-ray made suspect our diagnosis and it was confirmed intraoperatively. At 3 months postoperatively the patient has a strong abdominal wall. It is imperative to emphasize the importance of the physical examination goal of not losing diagnosis of TAWH.

## INTRODUCTION

Traumatic abdominal wall hernia (TAWH) is a rare clinical entity, with only a few cases reported since1906. Most cases are caused by an injury of falling on or hitting an angled or curved material, less common causes are high-energy type traumas like motorcycle accidents, fall from a height, seat belt injuries, pedestrian accidents and larger deceleration forces [1]. With few cases reported, a consensus in diagnosis and management has not been established in the literature [2]. The incidence of TAWH was found to be most prevalent in the male population younger than 50 years of age and presented as either ecchymosis (49%) or a localizing palpable hernia (31%) [2]. TAWH are often accompanied by intra-abdominal injuries [3]. Gastric injury is suspected following penetrating or blunt abdominal injury [4]. Diagnosis requires careful examination and high index of suspicion [5]. The presence of abdominal wall hematoma, abdominal wall tenderness, abrasion or ecchymosis may be the only findings [1]. Computed tomography (CT) scanning is crucial in the diagnosis of TAWH, and aids in definitive management of these patients. The literature supports immediate surgical exploration for most TAWH [2]. The management of the hernia is operative either with laparotomy or laparoscopy, primary or mesh repair should be appreciated according to size and site of the abdominal wall defect, coexisting intra-abdominal injury and timing of repair [6]. We treated a young patient man with a TAWH and gastric perforation after blunt abdominal trauma.

## CASE DESCRIPTION

A 20-year-old male, farmer presents to the emergency room of the hospital with 5 h of severe abdominal pain after he felt prone from a tractor impacting his abdomen against the stump of a felled tree; the pain was of sudden onset and constant and the site of the impact started to swell progressively. There was no personal history of abdominal wall hernias or chronic diseases.

**Figure 1:**
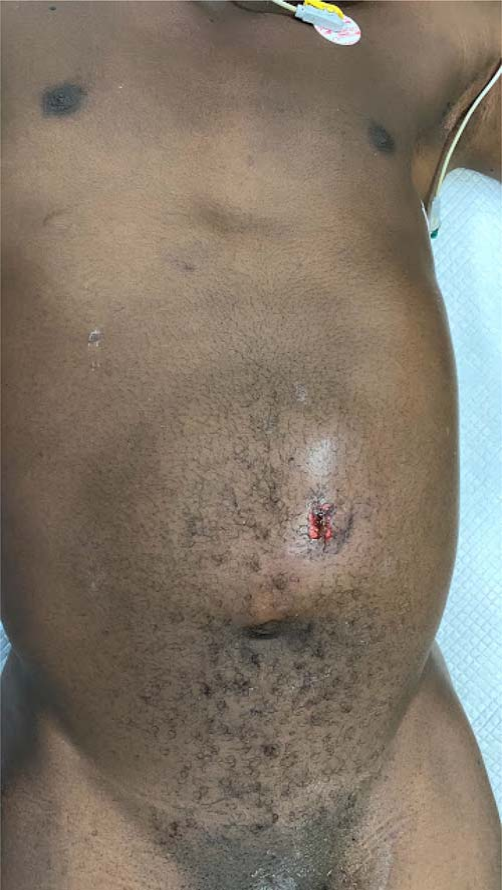
Anterior wall abdomen at presentation.

His physical examination revealed a slight abdominal distention with a swelling located above the umbilicus of ~10 by 10 cm with the presence of bruises and abrasions on the left side of the skin; the mass was partially reducible with the presence of subcutaneous emphysema extended to the left flank and hypochondrium (Fig. 1); the rest of the abdomen was very tender with board-like stiffness and rebound sensitivity; the pulse was 104, the BP was 150/90 MMHg. The complete blood count parameters were not remarkable, the erect chest radiograph showed pneumoperitoneum and the abdominal ultrasound free fluid in the cavity. Due to the economic limitation of the patient, we were unable to perform a CT scan of the abdomen. The presumptive diagnosis of TAWH with perforation of the hollow viscus was made preoperative.

**Figure 2:**
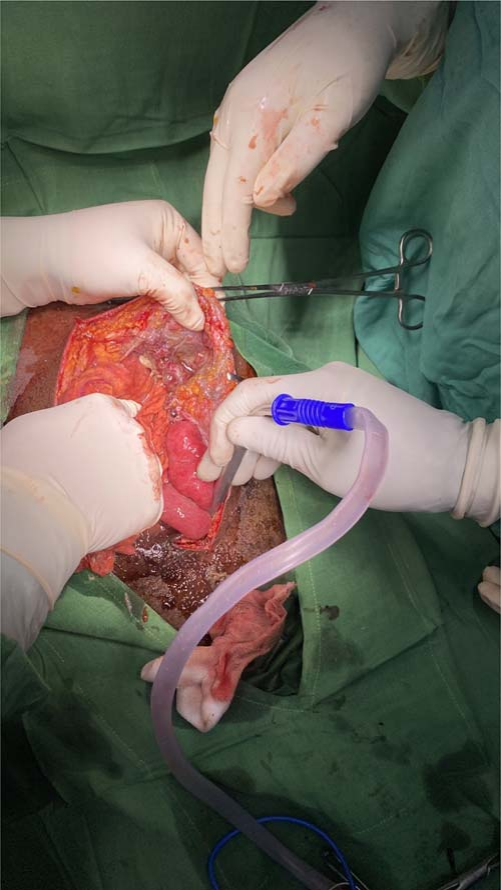
Fascia defect.

**Figure 3:**
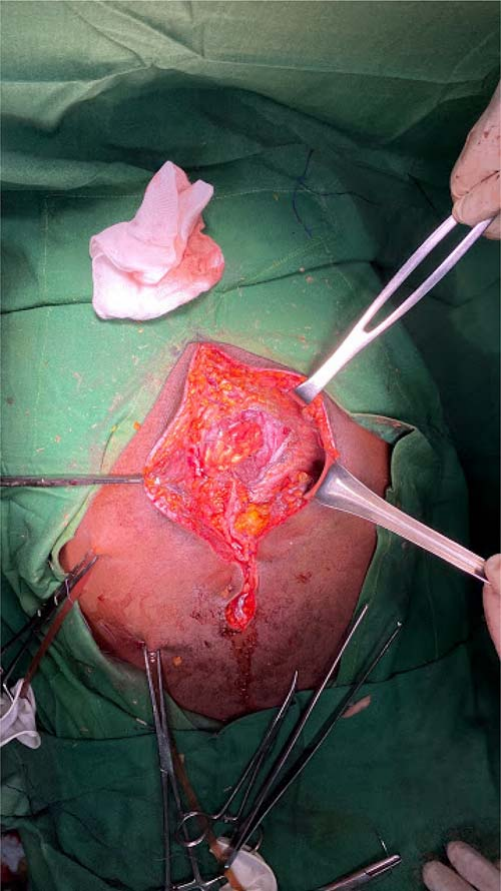
Obvious facial defect during repair

**Figure 4:**
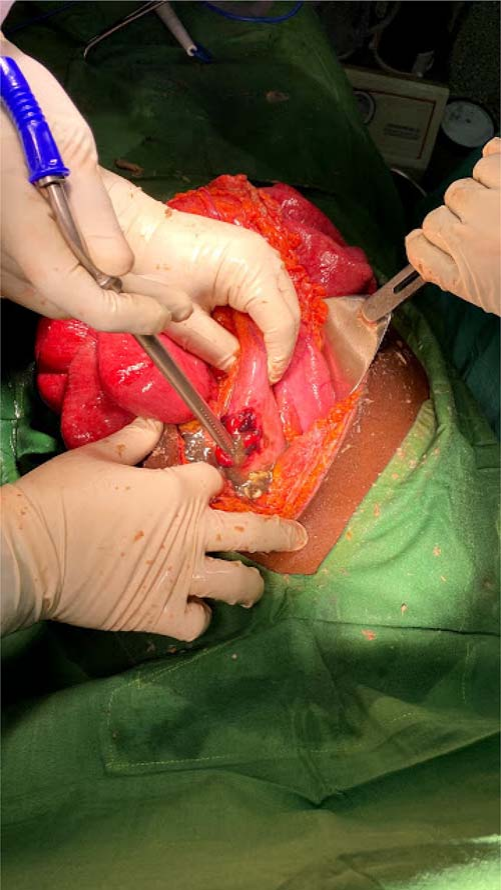
Gastric perforation.

**Figure 5:**
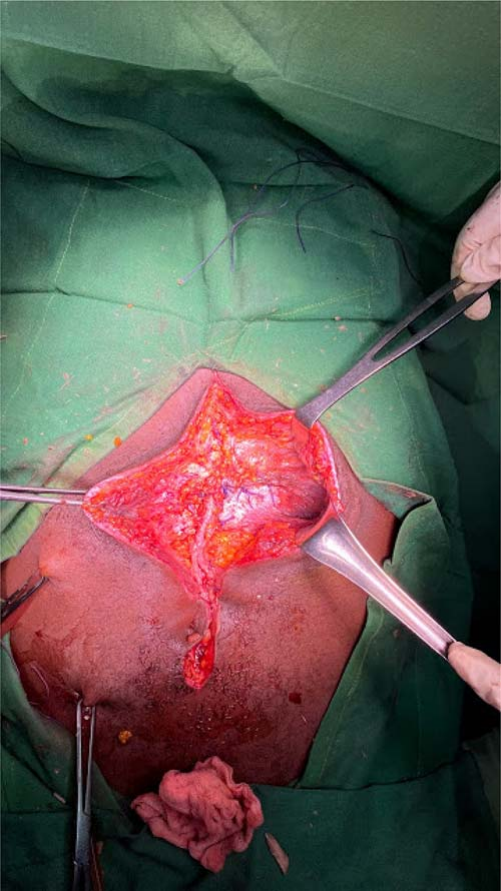
Fascia repaired.

**Figure 6:**
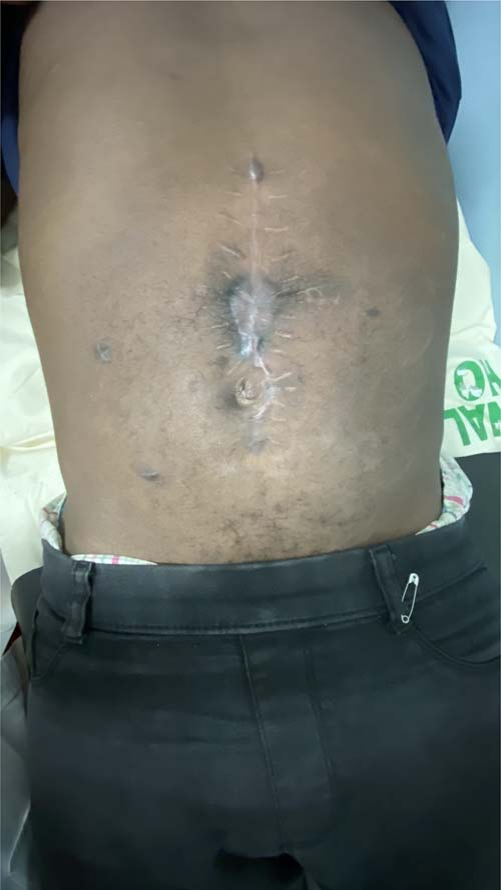
Anterior abdominal wall after 3 months of surgery.

An exploratory laparotomy was performed through an incision in the supra and infraumbilical midline, as we advanced we found food debris in the subcutaneous tissues and a defect with poorly defined borders of ~3 cm in the rectus muscle fascia with exposure of the abdominal content (Figs 2 and 3); A perforation was identified in the anterior aspect of the stomach towards the gastric body (Fig. 4) that was regularized and closed with a Gambee suture; primary repair of the fascia was performed with interrupted suture in the longitudinal and transverse planes (Fig. 5). There were no immediate postoperative complications; the Day 7 postoperative the patient presented a wound infection properly treated and was discharged on Day 12 followed in outpatient consultation with a strong abdominal wall (Fig. 6).

## DISCUSSION

In the literature, there is consensus that this kind of hernia occurs at anatomically weak points of the abdomen with a blunt trauma. An obvious fascia defect may exist with or without reducible hernia [1] and it is seen in <1.5% of blunt abdominal trauma patients [7, 8]. Differential diagnosis includes rectus sheath hematoma, tumor or pre-existing hernia; clinical survey is related not only to the hernia itself, but rather to associated or coexisting injuries; TAWH is rarely isolated; associated intra-abdominal injuries are determined in up to two-thirds of patients with TWAH and have been reported with an incidence between 25 and 70% [1, 8]. Clinically apparent anterior TAWHs appear to have a high rate of associated injuries requiring urgent laparotomy [9]. The authors recommend a high level of clinical suspicion for TAWH in all patients with traumatic abdominal wall injuries [10]. The diagnosis of abdominal wall injuries is typically straight forward on CT, thus, it is important for the radiologist to identify abdominal wall injuries and their associated injuries on admission CT, as these injuries typically require surgical correction early in the course of their management [9]. We present the case of a patient with an TAWH associated with gastric perforation after a blunt abdominal trauma in which the high level of suspicion was what led us to the diagnosis because it could not be confirmed by CT due to the low socio-economic situation of our patient.

The appropriate timing and approach of surgical treatment for TAWH depend on a case-by-case basis and represent a diagnostic and therapeutic challenge [11]. Open surgical repair in layers [10] or laparoscopic suturing during diagnostic laparoscopy may be appropriate in managing TAWH [12]. We did open primary closure of the fascia with interrupted suturing.

## CONCLUSION

TAWH should be suspected when patients present with an abdominal swelling following blunt abdominal trauma especially high-energy injuries to the abdomen. A good history and physical examination are of exceptional value in making an early diagnosis of traumatic hernia, being a challenge if there are concomitant intra-abdominal injuries where the use of CT scan will confirm it and help with the surgical management decision. Management typically involves surgical intervention to prevent complications.
